# The cost of keeping patients waiting: retrospective treatment-control study of additional healthcare utilisation for UK patients awaiting elective treatment

**DOI:** 10.1186/s12913-024-10931-2

**Published:** 2024-04-30

**Authors:** Charlotte James, Rachel Denholm, Richard Wood

**Affiliations:** 1 The National Institute for Health and Care Research Bristol Biomedical Research Centre (NIHR Bristol BRC), University Hospitals Bristol and Weston NHS Foundation Trust and University of Bristol, Bristol, UK; 2 The National Institute for Health and Care Research Applied Research Collaboration West (NIHR ARC West) at University Hospitals Bristol and Weston NHS Foundation Trust, Bristol, UK; 3 Population Health Sciences, Bristol Medical School, Bristol, UK; 4 Modelling and Analytics, UK National Health Service (BNSSG ICB), Bristol, UK; 5 Health Data Research UK South West, Bristol, UK; 6 Centre for Healthcare Innovation and Improvement (CHI2), School of Management, University of Bath, Bath, UK

**Keywords:** Elective recovery, Waitlist, Waiting times, Utilisation, Failure-demand

## Abstract

**Objective:**

Long waiting times for elective hospital treatments are common in many countries. This study seeks to address a deficit in the literature concerning the effect of long waits on the wider consumption of healthcare resources.

**Methods:**

We carried out a retrospective treatment-control study in a healthcare system in South West England from 15 June 2021 to 15 December 2021. We compared weekly contacts with health services of patients waiting over 18 weeks for treatment (‘Treatments’) and people not on a waiting list (‘Controls’). Controls were matched to Treatments based on age, sex, deprivation and multimorbidity. Treatments were stratified by the clinical specialty of the awaited hospital treatment, with healthcare usage assessed over various healthcare settings. Wilcoxon signed-rank tests assessed whether there was an increase in healthcare utilisation and bootstrap resampling was used to estimate the magnitude of any differences.

**Results:**

A total of 44,616 patients were waiting over 18 weeks (the constitutional target in England) for treatment during the study period. There was an increase (*p* < 0.0004) in healthcare utilisation for all specialties. Patients in the Cardiothoracic Surgery specialty had the largest increase, with 17.9 [interquartile-range: 4.3, 33.8] additional contacts with secondary care and 17.3 [-1.1, 34.1] additional prescriptions per year.

**Conclusion:**

People waiting for treatment consume higher levels of healthcare than comparable individuals not on a waiting list. These findings are relevant for clinicians and managers in better understanding patient need and reducing harm. Results also highlight the possible ‘false economy’ in failing to promptly resolve long elective waits.

**Supplementary Information:**

The online version contains supplementary material available at 10.1186/s12913-024-10931-2.

## Background

In the United Kingdom (UK), rising demand for healthcare has led to consistent increases in the size of waiting lists since 2014. As elective procedures were postponed during the pandemic, COVID-19 has accelerated this growth resulting in a substantial backlog of patients awaiting treatment. In July 2023, there were 7.7 million people in the UK waiting for treatment, an increase of over 50% compared to March 2020 [1]. 

The UK government announced an additional £8 billion over 3 years (2022–2025), allocated to the National Health Service (NHS) to tackle this backlog [2, 3]. For the purpose of decreasing waiting list size, this additional resource could be invested in: alternative methods for prioritising patients waiting for treatment [4]; pooling patients into larger surgical regions [5]; increasing capacity via improving surgical schedules, recruitment or use of the private sector [6, 7]. 

Alongside waiting list size, the median waiting time increased by more than 3 weeks during the COVID-19 pandemic [1]. In the UK there is an 18-week referral-to-treatment target [8]. Pre COVID, this target was being met for 86% of patients, however by March 2022 this had dropped to 62% [1]. For NHS services to optimise the value of interventions for tackling the waiting list backlog, not only does the resourcing of procedures need to be considered but also the additional resources consumed by people waiting for treatment.

The objective of this study is to quantify the difference in healthcare utilisation of people waiting over 18 weeks for treatment compared to a matched population not on a waiting list. Our results represent a first step in quantifying the hidden costs of keeping people waiting for treatment which is needed for determining care needs, minimising harm, and supporting future strategic planning.

## Methods

### Setting, design and population

We conducted a retrospective observational cohort study in Bristol, North Somerset and South Gloucestershire (BNSSG) Integrated Care System (ICS), which serves an approximate one million resident population in South West England across a mixture of urban, rural and coastal communities, with an overall age profile similar to that of England. The treatment group consisted of patients registered with a GP practice in BNSSG that were waiting over 18 weeks for treatment at any point in the period 15th June 2021 to 15th December 2021.

### Data

#### Referral to treatment

Referral to Treatment (RTT) data was obtained from NHS Digital via BNSSG ICS as a Commissioning Data Set [9, 10]. Since 2007, this data has been submitted by healthcare providers to NHS Digital Secondary Uses Service (SUS) each month. SUS are responsible for collating the data which is used by health care providers and commissioners for service planning and evaluation [9]. 

The RTT data represents all patients referred for consultant-led elective care to 18 main specialties: General Surgery; Urology; Trauma and Orthopaedic; Ear Nose and Throat; Ophthalmology; Oral Surgery; Neurosurgical; Plastic Surgery; Cardiothoracic Surgery; General Internal Medicine; Gastroenterology; Cardiology; Dermatology; Respiratory Medicine; Neurology; Rheumatology; Elderly Medicine and Gynaecology. Referrals to specialties outside of these 18 are grouped under ‘Other’ and not included in this analysis. The data includes date of referral, specialty, pathway status (open or closed) and date treatment started for each patient pathway. In the context of RTT data, treatment starting is the first appointment with a specialist consultant.

### Attributes and activity

Patient level data was obtained from the BNSSG System Wide Dataset (SWD) [11]. This dataset, which provides linkable data for 98.3% of the 1.07 million population of BNSSG, has been available since 2019. It contains two tables. The attributes table, generated from primary care data, is updated monthly and contains each person’s current demographic, socio-economic and clinical characteristics. The activity table contains information for all discrete patient contacts over the range of healthcare (points of delivery) within BNSSG ICS: Primary Care; Secondary Care; Helpline calls (the NHS 111 service); Emergency calls (the NHS 999 service); Community Services; Mental Health. Information is linked and pseudonymised by a third party, the NHS Commissioning Support Unit. Person Attributes and Activity are linkable through a unique patient identifier; a pseudonymised version of the NHS number. Data sources and descriptions are outlined in Table 1.

**Table 1:** Data sources used in the study

Point of delivery	Data source	Provider	Description
Primary care	EMIS GP administration system	OneCare	Bespoke extract contains data on GP attendances and prescriptions
Secondary care	Secondary use services	NHS Digital	All NHS acute trust outpatient consultations, inpatient admissions, and emergency department attendances
Mental health	Mental health services dataset	NHS Digital	Community mental health consultations and admitted stays in mental health hospitals
Community	Community services dataset	NHS Digital	Intermediate care admissions and patient visits to and from community service teams.
Emergency calls	SWAST	SWAST	Calls to 999
Helpline calls	Practice plus group	Practice plus group	Calls to 111

EMIS: Egton Medical Information Systems; GP: General Practitioner; SWAST: South West Ambulance Service

Study covariates were obtained from the attributes table and included age, sex, socioeconomic status, and presence of chronic conditions. Socioeconomic status was measured using the Index of Multiple Deprivation (IMD). IMD quantifies the relative deprivation of geographical areas in England. In the SWD, the IMD of a person corresponds to the IMD of the area they live in. Presence of chronic conditions was measured using the Quality and Outcomes Framework (QOF) indicators. These indicators include 20 conditions that lead to a person requiring higher levels of healthcare: atrial fibrillation; coronary heart disease; heart failure; hypertension; peripheral arterial disease; stroke; asthma; chronic obstructive pulmonary disease; obesity; cancer; chronic kidney disease; diabetes; palliative care; dementia; depression; epilepsy; learning disabilities; mental health conditions; osteoporosis; arthritis.

### Processing

For each person on one of the 18 waiting lists during the study period, a pool of controls was obtained from the SWD. Controls were randomly selected from the general population and matched to those on a waiting list, based on sex, 5-year age band, IMD quantile and individual QOF indicators (see above). Controls were excluded if they were on any waiting list during the study period.

For the treatment group, an individual study period was derived for each patient based on the time of referral and start of treatment (closed pathways). For the treatment group, follow up began at time of referral + 18 weeks, or study start date if already waiting > 18 weeks, and censored at date treatment started or end of the study period.

Mean activity per week, stratified by point of delivery, was determined for each participant. Activity was defined as a contact with each point of delivery, for example a GP appointment, a helpline call, etc. For the treatment group, activity directly associated with the referral, namely the GP appointment on the date of referral and the first outpatient appointment, i.e. the appointment associated with treatment starting, was not included in the analysis.

Patients in the treatment group can have more than one referral to a waiting list and be on waiting lists for multiple specialties. Where a patient had multiple referrals to one specialty, the first referral to a wait list was used, and start of treatment was defined as when all referrals were closed (i.e. the patient was no longer on the waiting list for that specialty). Specialities were analysed separately, as one patient could be on more than one waiting list.

### Statistical analysis

#### Is there a difference in healthcare utilisation?

A one-sided Wilcoxon signed-rank test was used to determine whether there was a difference in mean activity between the treatment and control groups. Given the many control candidates for each patient on a waiting list (and the desire to avoid reliance on a single selection), repeat bootstrap sampling (1,000 times, with replacement) was used to produce a multitude of treatment-control pairs for each analysis. The Wilcoxon signed-rank test was performed to compare the mean weekly activity of the treatment with the control group. For each of the 18 services and 7 points of delivery this yielded 1,000 p-values. A median p-value of less than 0.0004 provided strong evidence of a difference in the mean weekly activity of treatments compared to controls. This threshold was obtained by applying the Bonferroni correction for 126 statistical tests to a p-value of 0.05. A 95% confidence interval was obtained by taking the 2.5% and 97.5% percentiles of the 1,000 calculated p-values.

### What is the difference in healthcare utilisation?

To quantify the amount of additional healthcare utilisation we estimated the average difference in mean weekly healthcare contacts between the treatment and control group. We used repeat bootstrap sampling (1,000 times, with replacement). During each iteration we found the difference in mean weekly activity of each patient on the waiting list and a matched control. The median and inter-quartile range (IQR) of the distribution of differences was found for all treatment-control pairs, for each point of delivery and each specialty. Repeating this process 1,000 times resulted in distributions of median and IQR values. To summarise the amount of additional healthcare utilisation we reported the means of these distributions.

## Results

### Population characteristics

#### Treatment group

Of the 49,692 patients waiting over 18 weeks for treatment during the study period, 5076 had no matched controls and were excluded from the analysis. These patients had higher levels of multimorbidity, as measured by number of QOF conditions (Figure S1).

The final population consisted of 44,616 treatments across 18 specialties. Treatment characteristics for each speciality are shown in Table 2. The number of patients on each waiting list varies from 29 for Cardiothoracic Surgery to 6,889 for Trauma and Orthopaedic.

Most services have an approximately equal number of male and female treatments, however there are some exceptions: Gynaecology and Rheumatology are predominantly female whereas the Urology Service is predominantly male (100%, 71% and 30% female respectively). The mean age of the treatment groups is 53 +/- 5 (mean +/- standard deviation) years for all services apart from Oral Surgery and Gynaecology where patients tend to be younger (31 +/- 21 and 42 +/- 15 years respectively) and Elderly Medicine which has a mean age of 77 +/- 8 years. Dermatology had the lowest percentage of patients who were from a low socio-economic background (IMD < 4 = 7.9%), whilst oral surgery had the highest (33.8%). The mean number of QOF conditions is lowest for Oral Surgery (0.51 +/- 0.86) and highest for Elderly Medicine (1.67 +/- 1.34) reflecting the age difference of treatments across specialties. The number of controls per treatment is also reflective of this age difference, with Oral Surgery having the greatest (756.32 +/- 387.53) and Elderly Medicine having the least (200.38 +/- 334.82).

**Table 2:** Treatment description, stratified by specialty

Wait List	N	Female (%)	Age (Mean[SD])	IMD < 4 (%)	QOF (Mean[SD])	N Controls (Mean[SD])
Cardiology	2450	47.8	56.03 (18.44)	21.71	1.39 (1.25)	356.12 (430.01)
Cardiothoracic surgery	29	48.28	49.24 (24.2)	24.14	1.0 (0.95)	427.21 (453.25)
Dermatology service	1848	58.44	49.12 (22.38)	17.91	0.83 (1.06)	593.53 (437.5)
Ear nose and throat	3337	58.02	49.99 (19.94)	24.36	0.84 (1.04)	579.81 (438.18)
Elderly medicine	97	49.48	77.0 (7.69)	18.56	1.67 (1.34)	200.38 (334.82)
Gastroenterology	2185	50.98	53.23 (17.38)	24.58	1.06 (1.12)	494.29 (442.42)
General internal medicine	350	53.14	49.9 (18.9)	21.71	1.19 (1.18)	428.0 (447.82)
General surgery	4335	61.96	53.51 (17.29)	22.33	1.04 (1.17)	516.13 (447.12)
Gynaecology	4375	99.5	41.56 (15.07)	25.69	0.67 (0.87)	694.41 (400.39)
Neurology	1780	59.49	46.48 (18.2)	27.36	0.99 (1.05)	507.24 (447.25)
Neurosurgical	555	56.22	56.45 (16.07)	24.14	1.09 (1.13)	476.44 (437.39)
Ophthalmology	5718	58.17	65.15 (18.71)	21.3	1.28 (1.23)	370.32 (419.9)
Oral surgery	4200	55.93	31.01 (20.74)	33.83	0.51 (0.86)	756.32 (387.53)
Plastic surgery	930	56.77	51.99 (20.9)	21.18	0.84 (1.02)	563.87 (442.09)
Respiratory medicine	1342	47.17	54.22 (17.18)	26.23	1.46 (1.18)	321.3 (404.62)
Rheumatology	1018	71.12	49.71 (17.63)	30.26	0.99 (1.05)	513.62 (442.83)
Trauma and orthopaedic	6889	53.94	56.16 (18.0)	22.44	1.08 (1.14)	477.08 (440.81)
Urology	3178	29.61	58.11 (18.79)	22.34	1.14 (1.18)	446.07 (436.18)

IMD = Index of Multiple Deprivation. QOF = Quality and Outcomes Framework

### Healthcare utilisation

Across the 7 points of delivery, healthcare utilisation in the treatment group is greatest for primary care, prescriptions, and secondary care (Table 3). Patients waiting for Cardiothoracic Surgery have the greatest utilisation with a median of 3 [IQR: 1, 7.5] contacts with primary care, 15 [3, 33.5] prescriptions and 5.5 [2.25, 9] contacts with secondary care. For community services, mental health, helpline calls and emergency calls, the overall number of contacts during a treatment’s individual study period were very low: the median number of contacts is 0 for all four points of delivery (Table 3). Controls had lower healthcare utilisation during the study periods, with prescriptions being the only point of delivery where the median number of contacts is non-zero (Table 3).

**Table 3:** Number of contacts patients waiting over 18 weeks for treatment (treatments) and matched controls (controls) had with the health service during their individual study periods

Wait List	Controls				Treatments			
	Mean	Median	Std	IQR	Mean	Median	Std	IQR
	**Community**							
Cardiology service	0.06	0	0.87	[0,0]	0.63	0	5.62	[0,0]
Cardiothoracic surgery service	0.19	0	1.29	[0,0]	1.33	0	3.55	[0,0]
Dermatology service	0.05	0	0.71	[0,0]	0.46	0	4.73	[0,0]
Ear nose and throat service	0.09	0	1.11	[0,0]	0.50	0	5.63	[0,0]
Elderly medicine service	0.11	0	1.69	[0,0]	3.06	0	10.12	[0,0]
Gastroenterology service	0.06	0	1.12	[0,0]	0.49	0	3.48	[0,0]
General internal medicine service	0.06	0	0.93	[0,0]	0.99	0	4.53	[0,0]
General surgery service	0.07	0	1.09	[0,0]	1.19	0	7.84	[0,0]
Gynaecology service	0.08	0	0.84	[0,0]	0.34	0	2.88	[0,0]
Neurology service	0.05	0	0.69	[0,0]	0.90	0	7.04	[0,0]
Neurosurgical service	0.07	0	1.08	[0,0]	1.54	0	7.60	[0,0]
Ophthalmology service	0.10	0	1.60	[0,0]	1.02	0	9.18	[0,0]
Oral surgery service	0.10	0	0.74	[0,0]	0.24	0	2.72	[0,0]
Plastic surgery service	0.07	0	1.06	[0,0]	0.73	0	8.16	[0,0]
Respiratory medicine service	0.06	0	1.01	[0,0]	0.65	0	4.25	[0,0]
Rheumatology service	0.06	0	0.93	[0,0]	0.49	0	4.85	[0,0]
Trauma and orthopaedic service	0.07	0	1.26	[0,0]	0.86	0	7.51	[0,0]
Urology service	0.07	0	1.28	[0,0]	1.25	0	9.32	[0,0]
	**Mental Health**							
Cardiology service	0.06	0	0.82	[0,0]	0.17	0	1.87	[0,0]
Cardiothoracic surgery service	0.11	0	1.31	[0,0]	0.13	0	0.72	[0,0]
Dermatology service	0.07	0	0.83	[0,0]	0.12	0	0.93	[0,0]
Ear nose and throat service	0.09	0	1.02	[0,0]	0.24	0	1.76	[0,0]
Elderly medicine service	0.02	0	0.44	[0,0]	0.38	0	2.59	[0,0]
Gastroenterology service	0.06	0	0.80	[0,0]	0.23	0	2.36	[0,0]
General internal medicine service	0.06	0	0.79	[0,0]	0.26	0	2.24	[0,0]
General surgery service	0.06	0	0.80	[0,0]	0.20	0	1.75	[0,0]
Gynaecology service	0.11	0	1.09	[0,0]	0.20	0	1.34	[0,0]
Neurology service	0.08	0	0.92	[0,0]	0.50	0	3.13	[0,0]
Neurosurgical service	0.05	0	0.77	[0,0]	0.22	0	1.44	[0,0]
Ophthalmology service	0.04	0	0.68	[0,0]	0.13	0	1.35	[0,0]
Oral surgery service	0.08	0	0.98	[0,0]	0.21	0	1.80	[0,0]
Plastic surgery service	0.06	0	0.87	[0,0]	0.12	0	1.02	[0,0]
Respiratory medicine service	0.05	0	0.72	[0,0]	0.20	0	1.68	[0,0]
Rheumatology service	0.08	0	0.91	[0,0]	0.28	0	1.98	[0,0]
Trauma and orthopaedic service	0.06	0	0.85	[0,0]	0.16	0	1.50	[0,0]
Urology service	0.05	0	0.76	[0,0]	0.19	0	1.71	[0,0]
	**Emergency Calls**							
Cardiology service	0.01	0	0.15	[0,0]	0.11	0	0.52	[0,0]
Cardiothoracic surgery service	0.03	0	0.21	[0,0]	0.23	0	0.56	[0,0]
Dermatology service	0.01	0	0.14	[0,0]	0.04	0	0.27	[0,0]
Ear nose and throat service	0.02	0	0.18	[0,0]	0.08	0	0.49	[0,0]
Elderly medicine service	0.02	0	0.14	[0,0]	0.22	0	0.71	[0,0]
Gastroenterology service	0.01	0	0.16	[0,0]	0.19	0	2.18	[0,0]
General internal medicine service	0.01	0	0.16	[0,0]	0.11	0	0.43	[0,0]
General surgery service	0.01	0	0.16	[0,0]	0.10	0	0.50	[0,0]
Gynaecology service	0.02	0	0.19	[0,0]	0.07	0	0.58	[0,0]
Neurology service	0.01	0	0.16	[0,0]	0.22	0	1.74	[0,0]
Neurosurgical service	0.01	0	0.16	[0,0]	0.15	0	0.69	[0,0]
Ophthalmology service	0.01	0	0.16	[0,0]	0.08	0	0.38	[0,0]
Oral surgery service	0.02	0	0.17	[0,0]	0.04	0	0.26	[0,0]
Plastic surgery service	0.01	0	0.16	[0,0]	0.05	0	0.29	[0,0]
Respiratory medicine service	0.01	0	0.16	[0,0]	0.11	0	0.44	[0,0]
Rheumatology service	0.01	0	0.16	[0,0]	0.07	0	0.37	[0,0]
Trauma and orthopaedic service	0.02	0	0.17	[0,0]	0.08	0	0.45	[0,0]
Urology service	0.01	0	0.16	[0,0]	0.12	0	0.76	[0,0]
	**Helpline Calls**							
Cardiology service	0.03	0	0.20	[0,0]	0.09	0	0.45	[0,0]
Cardiothoracic surgery service	0.06	0	0.33	[0,0]	0.07	0	0.36	[0,0]
Dermatology service	0.03	0	0.21	[0,0]	0.05	0	0.26	[0,0]
Ear nose and throat service	0.04	0	0.28	[0,0]	0.09	0	0.41	[0,0]
Elderly medicine service	0.01	0	0.14	[0,0]	0.12	0	0.45	[0,0]
Gastroenterology service	0.03	0	0.24	[0,0]	0.11	0	0.68	[0,0]
General internal medicine service	0.03	0	0.22	[0,0]	0.12	0	0.56	[0,0]
General surgery service	0.03	0	0.26	[0,0]	0.09	0	0.47	[0,0]
Gynaecology service	0.04	0	0.31	[0,0]	0.12	0	0.79	[0,0]
Neurology service	0.03	0	0.27	[0,0]	0.15	0	0.74	[0,0]
Neurosurgical service	0.03	0	0.21	[0,0]	0.11	0	0.49	[0,0]
Ophthalmology service	0.03	0	0.20	[0,0]	0.07	0	0.35	[0,0]
Oral surgery service	0.05	0	0.30	[0,0]	0.08	0	0.38	[0,0]
Plastic surgery service	0.03	0	0.28	[0,0]	0.07	0	0.36	[0,0]
Respiratory medicine service	0.03	0	0.21	[0,0]	0.09	0	0.40	[0,0]
Rheumatology service	0.03	0	0.26	[0,0]	0.07	0	0.32	[0,0]
Trauma and orthopaedic service	0.03	0	0.26	[0,0]	0.09	0	0.54	[0,0]
Urology service	0.03	0	0.22	[0,0]	0.12	0	0.60	[0,0]
	**Primary Care**							
Cardiology service	1.00	0	2.21	[0,1]	3.33	1	5.10	[0,5]
Cardiothoracic surgery service	1.44	0	2.69	[0,2]	5.90	3	7.17	[1,7.5]
Dermatology service	0.86	0	1.96	[0,1]	2.47	1	4.20	[0,3]
Ear nose and throat service	1.40	0	2.66	[0,2]	3.84	2	5.06	[0,5]
elderly medicine service	1.26	0	2.30	[0,2]	3.53	2	5.07	[0,5]
Gastroenterology service	1.10	0	2.35	[0,1]	3.56	2	5.27	[0,5]
General internal medicine service	1.11	0	2.40	[0,1]	4.24	2	6.16	[0,6]
General surgery service	1.14	0	2.39	[0,1]	4.05	2	6.58	[0,5]
Gynaecology service	1.30	0	2.56	[0,2]	3.09	1	4.69	[0,4]
Neurology service	0.97	0	2.20	[0,1]	3.24	1	5.25	[0,4]
Neurosurgical service	1.14	0	2.38	[0,1]	4.12	2	5.59	[0,6]
Ophthalmology service	1.17	0	2.38	[0,1]	3.20	1	5.13	[0,4]
Oral surgery service	0.90	0	2.06	[0,1]	1.81	0	3.44	[0,2]
Plastic surgery service	1.00	0	2.21	[0,1]	2.96	1	5.74	[0,4]
Respiratory medicine service	1.10	0	2.33	[0,1]	3.99	2	5.48	[0,6]
Rheumatology service	1.07	0	2.32	[0,1]	3.22	1	4.48	[0,4]
Trauma and orthopaedic service	1.26	0	2.54	[0,2]	4.14	2	6.04	[0,6]
Urology service	1.11	0	2.39	[0,1]	4.01	2	5.58	[0,6]
	**Prescription**							
Cardiology service	2.08	1	5.77	[0,2]	14.51	5	32.10	[1,16]
Cardiothoracic surgery service	2.31	1	5.89	[0,2]	18.13	15	15.47	[3,33.5]
Dermatology service	1.51	0	4.40	[0,2]	6.99	2	16.98	[0,7]
Ear nose and throat service	2.70	1	7.04	[0,3]	12.30	4	29.38	[1,13]
Elderly medicine service	3.66	1	7.76	[0,4]	19.56	12	29.55	[2,26]
Gastroenterology service	2.26	1	6.33	[0,2]	14.11	5	32.00	[1,15]
General internal medicine service	2.30	1	6.44	[0,2]	17.67	7	39.48	[1,17]
General surgery service	2.27	1	6.33	[0,2]	14.17	4	34.56	[1,14]
Gynaecology service	2.00	1	5.86	[0,2]	6.67	2	16.92	[0,6]
Neurology service	1.67	0	4.97	[0,2]	11.81	3	32.25	[0,11]
Neurosurgical service	2.55	1	7.12	[0,2]	19.44	7	43.53	[1,19]
Ophthalmology service	2.87	1	7.28	[0,3]	15.07	6	32.11	[1,16]
Oral surgery service	1.22	0	4.09	[0,1]	4.77	1	16.02	[0,3]
Plastic surgery service	1.90	0	5.60	[0,2]	9.00	2	20.21	[0,9]
Respiratory medicine service	2.40	1	6.32	[0,2]	16.56	7	31.47	[1,20]
Rheumatology service	2.01	1	5.72	[0,2]	11.70	4	25.73	[0,12]
Trauma and orthopaedic service	2.80	1	7.41	[0,3]	16.14	6	34.26	[1,19]
Urology service	2.60	1	6.92	[0,2]	16.90	6	35.34	[1,19]
	**Secondary Care**							
Cardiology service	0.30	0	1.50	[0,0]	1.99	1	5.21	[0,2]
Cardiothoracic surgery service	0.44	0	2.00	[0,0]	8.07	5.5	10.54	[2.3,9]
Dermatology service	0.27	0	1.39	[0,0]	1.25	0	2.88	[0,1]
Ear nose and throat service	0.43	0	1.88	[0,0]	2.13	1	4.01	[0,3]
Elderly medicine service	0.34	0	1.40	[0,0]	2.18	1	3.41	[0,3]
Gastroenterology service	0.31	0	1.53	[0,0]	2.58	1	5.34	[0,3]
General internal medicine service	0.31	0	1.50	[0,0]	3.22	2	6.19	[0,4]
General surgery service	0.33	0	1.62	[0,0]	2.94	1	6.61	[0,3]
Gynaecology service	0.45	0	2.06	[0,0]	2.06	1	3.96	[0,3]
Neurology service	0.30	0	1.52	[0,0]	1.96	0	4.27	[0,2]
Neurosurgical service	0.31	0	1.48	[0,0]	2.93	1	4.49	[0,4]
Ophthalmology Service	0.33	0	1.48	[0,0]	2.02	1	3.98	[0,3]
Oral surgery service	0.34	0	1.54	[0,0]	0.78	0	2.20	[0,1]
Plastic surgery service	0.29	0	1.46	[0,0]	2.27	1	4.43	[0,3]
Respiratory medicine service	0.30	0	1.44	[0,0]	2.95	2	4.73	[0,4]
Rheumatology service	0.33	0	1.63	[0,0]	1.94	0	3.69	[0,2]
Trauma and orthopaedic service	0.35	0	1.63	[0,0]	2.75	1	4.36	[0,4]
Urology service	0.31	0	1.49	[0,0]	2.76	1	4.93	[0,3.8]

### Is there a difference in healthcare utilisation?

There is evidence of a difference in the healthcare utilisation of patients waiting over 18 weeks for treatment compared to matched controls (Table S1). On average, patients waiting for treatment have more contacts with primary care, secondary care, helpline calls and prescriptions compared to those not waiting for treatment (*p* < 0.0004, ‘All Specialties’, Table S1). In all specialties, the secondary care utilisation of patients waiting for treatment is greater than matched controls (*p* < 0.0004). Cardiothoracic Surgery and Elderly Medicine are the only specialties for which there is little evidence of an increased use of primary care across the two groups (*p* = 0.0321 [0.0011, 0.2519] and *p* = 0.0024 [0.0001, 0.0463] respectively). There is strong evidence that patients waiting for the General Surgery, Urology, Neurology and Gastroenterology services show increased utilisation across all points of delivery (*p* < 0.0004), compared to controls. In contrast, patients waiting for Cardiothoracic Surgery show an increased utilisation in secondary care only.

### What is the difference in healthcare utilisation?

**Fig. 1:**
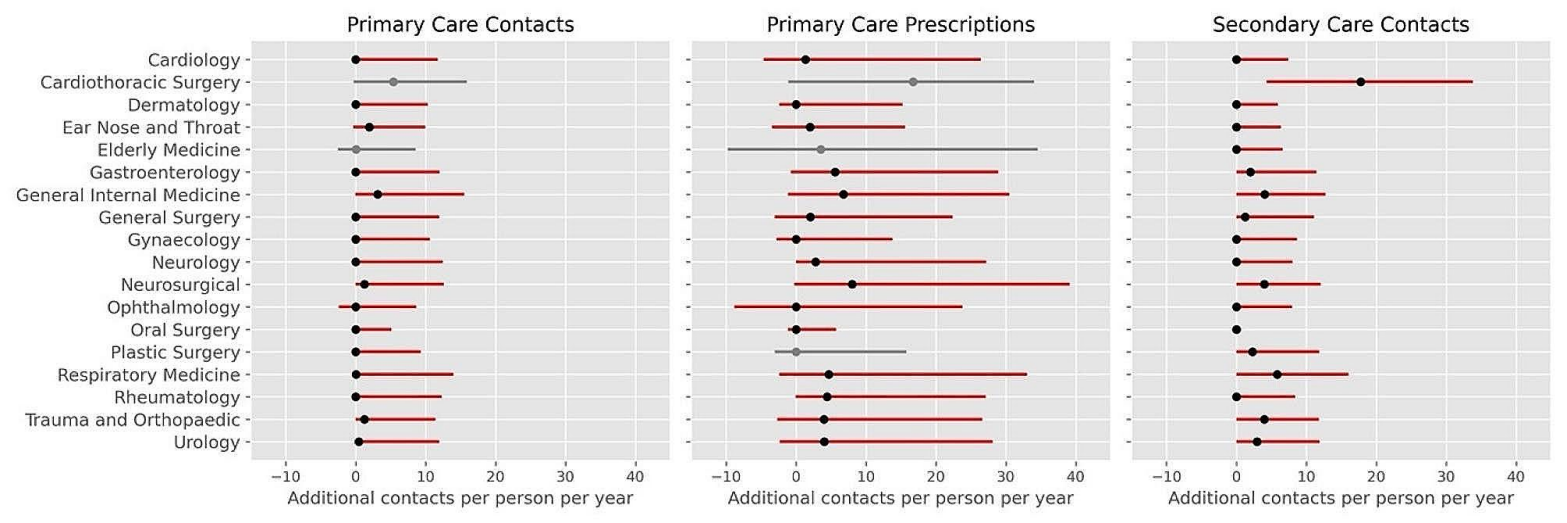
Additional yearly contacts with primary care, primary care prescriptions, and secondary care for patients waiting for treatment (treatments) compared to matched controls. Additional contacts per person per year were obtained by bootstrap sampling controls for each treatment 1000 times, resulting in 1000 distributions. Black dots represent the median additional contacts per person per year, obtained by averaging the median values of the 1000 distributions. Red lines represent the mean 25th percentile and 75th percentile (interquartile range) of the 1000 distributions of additional contacts, per person, per year. For specialties where there was no evidence of a difference in utilisation (*p* > 0.0004), results are displayed in grey

Patients waiting over 18 weeks for treatment demonstrate higher levels of healthcare utilisation than patients not waiting for treatment (Fig. 1, Table S2). The additional healthcare utilisation is greatest for primary care prescriptions, followed by secondary care. Patients waiting for the Cardiothoracic Surgery service have a median of 17.9 [4.3, 33.8] additional contacts with secondary care and 17.3 [-1.1, 34.1] additional prescriptions per year compared to matched controls not waiting for treatment. Patients waiting for this service also show the largest median increase in the number of GP appointments (‘primary care contact’) (5.5 [-0.3, 15.9]), however in this point of delivery at least 25% of people waiting for Cardiothoracic Surgery show no increase in utilisation (25th percentile < 0). Of the 18 specialties, patients waiting for the Oral Surgery, Ophthalmology, Gynaecology and Dermatology services had median increases of 0.0 across all points of delivery: at least 50% of patients waiting for these services had no additional healthcare utilisation in all points of delivery (Table S2). The helpline calls, emergency calls, community and mental health services show the smallest increases in healthcare utilisation (Table S2), with these services not used at all by more than 75% of patients in both groups. This suggests that the increased utilisation for some of these services (*p* < 0.0004) can be attributed to less than 25% of the people waiting for treatment.

## Discussion

Our study marks an initial step in quantifying the hidden cost of increases in waiting list size and waiting times. Using a retrospective cohort study design we have demonstrated that the healthcare utilisation of patients waiting over 18 weeks for treatment is greater than a matched population not waiting for treatment. We found evidence of increases in healthcare utilisation in each specialty and across all points of delivery, however the size of the differences varied: patients waiting for the Cardiothoracic Surgery service showed the greatest increase, while at least 50% of patients waiting for the Oral Surgery, Ophthalmology, Gynaecology and Dermatology services had no additional healthcare utilisation. The largest increases were seen across primary care prescriptions and secondary care. Mental health services, emergency calls and helpline calls had the smallest differences. Our results demonstrate the burden of increasing waiting times on both patients and health services.

We selected our control group as people not waiting for treatment, to quantify the additional healthcare utilisation due to patients not being seen within the NHS 18-week target. Our rationale for this choice of control group is that, if a patient starts treatment within the 18-week target, their healthcare need is being met. Once treatment is completed a patient’s healthcare need should be comparable to a similar person (in terms of our matching criteria) that hadn’t required treatment. In contrast, when a patient is waiting longer than the 18-week target, their need is not being met. By comparing the healthcare utilisation of people waiting over 18 weeks for treatment to people not waiting for treatment, we are estimating the additional burden on health services due to the inability to meet the 18-week target for many patients.

Treatments were matched to controls based on sex, 5-year age band, IMD quantile and twenty long-term conditions (QOF indicators). QOF indicators were included in the matching criteria to control for differences in baseline healthcare need between treatments and controls; many of these conditions require long-term management, the nature of which will vary between indicators, but will be evident in a patient’s health service utilisation. Although these matching criteria are stringent, it is not possible to conclude with certainty that the differences in health service utilisation seen in this study are caused by waiting over 18-weeks for treatment. In terms of comorbidities, the twenty QOF indicators included are not exhaustive, and patients waiting for treatment may have additional comorbidities that are not accounted for in the matching. In addition, the QOF indicators themselves may introduce confounding; although QOF indicators mark the presence of a diagnosis, they do not measure the severity of the underlying condition. Severity may be considered a confounder in our study; if patients are waiting for a treatment in relation to a QOF condition, this condition is likely to be more severe, and require greater levels of long-term management. However, had such a patient received treatment within the 18-week target, we would expect the difference in healthcare utilisation to be lower.

Our study population shows that the number of people waiting over 18 weeks for treatment varies between specialties (Table 2). This variation is likely due to differences in both demand and capacity across the specialties. As our analysis has been carried out in each specialty independently, these differences in population size need to be considered when comparing results between specialties. For example, patients waiting for Cardiothoracic Surgery show the greatest increase in secondary care utilisation (median 17.9 additional contacts per year, Table S2), however this specialty also has the smallest sample size (29 patients, Table 2), therefore in total this corresponds to approximately 520 additional contacts with secondary care per year. In contrast, patients waiting for Respiratory Medicine have, on average, fewer additional contacts with secondary care (median 5.9, Table S2) but as this population is larger (1342 patients, Table 2), this represents a higher burden on secondary care in this healthcare system: approximately 7900 additional contacts over the course of a year.

A consequence of the differences in population size across specialties is that, for some specialties, the population may represent low priority, less urgent referrals, who are waiting longer due to the prioritisation of more urgent cases. In contrast, for other specialties, the population may contain a mix of both urgent and non-urgent referrals, who are waiting over 18-weeks due to increased demand, reduced capacity, or both. This is a limitation of our study: we have not controlled for referral priority within the treatment groups, and more urgent cases are likely to have a higher healthcare need while waiting for treatment to start. However, although this affects comparisons between specialties, it should not affect specialty level results: within each specialty the population waiting over 18-weeks for treatment represents how wait lists are managed for that specialty, therefore our results represent what is typical for each specialty within the locality.

Our study period covers the six months immediately following the easing of restrictions in the UK that were in place due to the COVID-19 pandemic, and a time when UK policy was focussed on the recovery of elective services. Throughout the study period there were no further restrictions in place, however during the study period healthcare utilisation may still have been impacted by the pandemic; an analysis of private healthcare utilisation in the UK from January 2018 to August 2020 found, at the end of this period, utilisation had not returned to pre-pandemic levels [12]. On the one hand this may be seen as a limitation of our study; if healthcare utilisation had not returned to pre-pandemic levels during our study period, our results may overestimate the difference in activity between treatment and controls. However, by comparing activity differences of treatments and controls over weekly time-windows, our study design reduces the effect of longitudinal, population-level, changes in healthcare utilisation, therefore our estimates are unlikely to be impacted by changes in utilisation due to covid.

This is one of the first studies to consider the wider implications of long waiting times for patients and health services. From a value-based perspective, knowledge of the amount of extra resource being spent on patients while waiting for treatment is crucial to optimising the cost-effectiveness of any intervention to reduce waiting list size. The objective of our study was to determine whether there was an increase in healthcare utilisation when waiting for treatment. By quantifying the magnitude of this increase, our results will assist strategic planners in assigning a cost to waiting times and waiting list size.

The additional service utilisation of patients waiting over 18 weeks for treatment should be considered as an example of failure-demand within the health service: if patients received treatment earlier, they would not be requiring additional support over a prolonged period. Previous work has demonstrated how failure-demand generated by one component of a health system results in demand being deflected to other components [13, 14]. The consequence is an increase in pressure elsewhere in the system and a negative impact on patient experience [13]. In combination with our results, this highlights the need for a whole-system approach to tackling the waiting list backlog: a thorough evaluation of interventions, such as increasing surgical capacity and revising existing methods for prioritising patients, requires consideration of the impact on the health service as a whole.

## Conclusion

We have provided evidence that patients waiting for treatment in the UK have higher levels of healthcare utilisation than people not waiting for treatment. Our results can be used to evaluate the cost-effectiveness of interventions to reduce the waiting list backlog. The evidence of increased healthcare utilisation highlights the need for a whole-system approach to tackling the waiting list backlog.

### Electronic supplementary material

Below is the link to the electronic supplementary material.

Supplementary Material 1

## Data Availability

Data analysed during this study cannot be made available due to local restrictions on public sharing of patient-level information.

## Abbreviations

UK: United Kingdom
NHS: National Health Service
BNSSG: Bristol, North Somerset and South Gloucestershire
ICS: Integrated Care System
RTT: Referral to Treatment
SUS: Secondary Uses Service
SWD: System Wide Dataset
IMD: Index of Multiple Deprivation
QOF: Quality and Outcomes Framework
IQR: Inter-quartile range
