# Supplementary material for: The cost of keeping patients waiting: retrospective treatment-control study of additional healthcare utilisation for UK patients awaiting elective treatment
Source: BMC Health Serv Res. 2024 Apr 30;24:556. doi: 10.1186/s12913-024-10931-2 (PMC11061904; doi:10.1186/s12913-024-10931-2)
Supplement: Supplementary file 1 — Supplementary Material 1 [file 12913_2024_10931_MOESM1_ESM.docx]

**Supplement**

***Population characteristics***

***
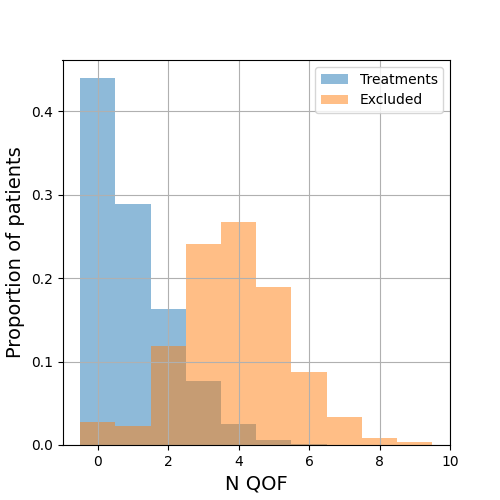
***

**Figure S1** Distribution of number of Quality and Outcomes Framework (QOF) conditions for treatments (blue) and patients excluded from the analysis due having to no matched controls (orange).

***Is there a difference in health service utilisation?***

| **Specialty** | **Helpline calls** | **Primary care contact** | **Primary care prescription** | **Community** | **Emergency calls** | **Secondary** | **Mental health** |
| --- | --- | --- | --- | --- | --- | --- | --- |
| Cardiology Service | 0.0000 [0.0000, 0.0000] | 0.0000 [0.0000, 0.0000] | 0.0000 [0.0000, 0.0000] | 0.0353 [0.0016, 0.2061] | 0.0000 [0.0000, 0.0000] | 0.0000 [0.0000, 0.0000] | 0.0145 [0.0002, 0.1805] |
| Cardiothoracic Surgery Service | 0.1244 [0.0216, 0.6168] | 0.0024 [0.0001, 0.0463] | 0.0079 [0.0004, 0.1008] | 0.0137 [0.0054, 0.1423] | 0.0865 [0.0213, 0.3994] | 0.0000 [0.0000, 0.0000] | 0.6425 [0.2965, 0.9279] |
| Dermatology Service | 0.0000 [0.0000, 0.0002] | 0.0000 [0.0000, 0.0000] | 0.0000 [0.0000, 0.0000] | 0.2686 [0.0393, 0.6906] | 0.1378 [0.0111, 0.5672] | 0.0000 [0.0000, 0.0000] | 0.0550 [0.0011, 0.4000] |
| Ear Nose and Throat Service | 0.0000 [0.0000, 0.0000] | 0.0000 [0.0000, 0.0000] | 0.0000 [0.0000, 0.0000] | 0.0646 [0.0022, 0.3798] | 0.0000 [0.0000, 0.0000] | 0.0000 [0.0000, 0.0000] | 0.0000 [0.0000, 0.0018] |
| Elderly Medicine Service | 0.0045 [0.0005, 0.0502] | 0.0321 [0.0011, 0.2519] | 0.0188 [0.0009, 0.1474] | 0.0001 [0.0000, 0.0054] | 0.0219 [0.0010, 0.1637] | 0.0000 [0.0000, 0.0000] | 0.0610 [0.0032, 0.3077] |
| Gastroenterology Service | 0.0000 [0.0000, 0.0000] | 0.0000 [0.0000, 0.0000] | 0.0000 [0.0000, 0.0000] | 0.0000 [0.0000, 0.0009] | 0.0000 [0.0000, 0.0000] | 0.0000 [0.0000, 0.0000] | 0.0002 [0.0000, 0.0136] |
| General Internal Medicine Service | 0.0126 [0.0004, 0.1239] | 0.0000 [0.0000, 0.0000] | 0.0000 [0.0000, 0.0000] | 0.0000 [0.0000, 0.0023] | 0.0007 [0.0000, 0.0208] | 0.0000 [0.0000, 0.0000] | 0.1876 [0.0149, 0.6369] |
| General Surgery Service | 0.0000 [0.0000, 0.0000] | 0.0000 [0.0000, 0.0000] | 0.0000 [0.0000, 0.0000] | 0.0000 [0.0000, 0.0000] | 0.0000 [0.0000, 0.0000] | 0.0000 [0.0000, 0.0000] | 0.0003 [0.0000, 0.0161] |
| Gynaecology Service | 0.0000 [0.0000, 0.0000] | 0.0000 [0.0000, 0.0000] | 0.0000 [0.0000, 0.0000] | 0.0043 [0.0000, 0.0998] | 0.0000 [0.0000, 0.0000] | 0.0000 [0.0000, 0.0000] | 0.0026 [0.0000, 0.0816] |
| Neurology Service | 0.0000 [0.0000, 0.0000] | 0.0000 [0.0000, 0.0000] | 0.0000 [0.0000, 0.0000] | 0.0000 [0.0000, 0.0000] | 0.0000 [0.0000, 0.0000] | 0.0000 [0.0000, 0.0000] | 0.0000 [0.0000, 0.0000] |
| Neurosurgical Service | 0.0000 [0.0000, 0.0003] | 0.0000 [0.0000, 0.0000] | 0.0000 [0.0000, 0.0000] | 0.0000 [0.0000, 0.0000] | 0.0000 [0.0000, 0.0000] | 0.0000 [0.0000, 0.0000] | 0.0041 [0.0000, 0.0597] |
| Ophthalmology Service | 0.0000 [0.0000, 0.0000] | 0.0000 [0.0000, 0.0000] | 0.0000 [0.0000, 0.0025] | 0.0074 [0.0001, 0.1323] | 0.0000 [0.0000, 0.0001] | 0.0000 [0.0000, 0.0000] | 0.0082 [0.0001, 0.1204] |
| Oral Surgery Service | 0.0000 [0.0000, 0.0000] | 0.0000 [0.0000, 0.0000] | 0.0000 [0.0000, 0.0000] | 0.5166 [0.0927, 0.9176] | 0.0000 [0.0000, 0.0050] | 0.0000 [0.0000, 0.0000] | 0.0007 [0.0000, 0.0387] |
| Plastic Surgery Service | 0.0023 [0.0000, 0.0567] | 0.0000 [0.0000, 0.0000] | 0.0000 [0.0000, 0.0009] | 0.0411 [0.0022, 0.2962] | 0.0991 [0.0070, 0.4516] | 0.0000 [0.0000, 0.0000] | 0.1174 [0.0077, 0.5143] |
| Respiratory Medicine Service | 0.0000 [0.0000, 0.0000] | 0.0000 [0.0000, 0.0000] | 0.0000 [0.0000, 0.0000] | 0.0000 [0.0000, 0.0002] | 0.0000 [0.0000, 0.0000] | 0.0000 [0.0000, 0.0000] | 0.0079 [0.0001, 0.1179] |
| Rheumatology Service | 0.0000 [0.0000, 0.0003] | 0.0000 [0.0000, 0.0000] | 0.0000 [0.0000, 0.0000] | 0.0015 [0.0000, 0.0381] | 0.0002 [0.0000, 0.0052] | 0.0000 [0.0000, 0.0000] | 0.0025 [0.0000, 0.0652] |
| Trauma and Orthopaedic Service | 0.0000 [0.0000, 0.0000] | 0.0000 [0.0000, 0.0000] | 0.0000 [0.0000, 0.0000] | 0.0000 [0.0000, 0.0000] | 0.0000 [0.0000, 0.0000] | 0.0000 [0.0000, 0.0000] | 0.0006 [0.0000, 0.0216] |
| Urology Service | 0.0000 [0.0000, 0.0000] | 0.0000 [0.0000, 0.0000] | 0.0000 [0.0000, 0.0000] | 0.0000 [0.0000, 0.0000] | 0.0000 [0.0000, 0.0000] | 0.0000 [0.0000, 0.0000] | 0.0000 [0.0000, 0.0023] |
| **All Specialties*** | 0.0002 [0.0000,0.0027] | 0.0001 [0.0000,0.0006] | 0.0000 [0.0000,0.0007] | 0.0688 [0.0107,0.1887] | 0.0079 [0.0006,0.0343] | 0.0000 [0.0000,0.0000] | 0.0094 [0.0006,0.0822] |

**Table S1** Results of bootstrapped Wilcoxon signed-rank tests to assess whether there is a significant difference in weekly activity of treatments and controls more than 18 weeks after a referral is made. Values correspond to median [CI] p-values. P-values < 0.0004 correspond to strong evidence of an increase in healthcare utilisation. *median [CI] p-values across all specialties, obtained via a weighted average of p-values for each specialty.

***How large is the difference in health service utilisation?***

|  | **Helpline Calls** | |
| --- | --- | --- |
| **Specialty** | **Median** | **IQR** |
| Cardiology Service | 0.0 | [0.0,0.0] |
| Cardiothoracic Surgery Service | 0.0 | [0.0,0.0] |
| Dermatology Service | 0.0 | [0.0,0.0] |
| Ear Nose and Throat Service | 0.0 | [0.0,0.0] |
| Elderly Medicine Service | 0.0 | [0.0,0.0] |
| Gastroenterology Service | 0.0 | [0.0,0.0] |
| General Internal Medicine Service | 0.0 | [0.0,0.0] |
| General Surgery Service | 0.0 | [0.0,0.0] |
| Gynaecology Service | 0.0 | [0.0,0.0] |
| Neurology Service | 0.0 | [0.0,0.0] |
| Neurosurgical Service | 0.0 | [0.0,0.0] |
| Ophthalmology Service | 0.0 | [0.0,0.0] |
| Oral Surgery Service | 0.0 | [0.0,0.0] |
| Plastic Surgery Service | 0.0 | [0.0,0.0] |
| Respiratory Medicine Service | 0.0 | [0.0,0.0] |
| Rheumatology Service | 0.0 | [0.0,0.0] |
| Trauma and Orthopaedic Service | 0.0 | [0.0,0.0] |
| Urology Service | 0.0 | [0.0,0.0] |
|  | **Primary Care Contact** | |
|  | **Median** | **IQR** |
| Cardiology Service | 0.0 | [0.0,11.8] |
| Cardiothoracic Surgery Service | 5.5 | [-0.3,15.9] |
| Dermatology Service | 0.0 | [0.0,10.3] |
| Ear Nose and Throat Service | 2.0 | [-0.3,10.0] |
| Elderly Medicine Service | 0.0 | [-2.5,8.6] |
| Gastroenterology Service | 0.0 | [0.0,12.0] |
| General Internal Medicine Service | 3.1 | [0.0,15.5] |
| General Surgery Service | 0.0 | [0.0,11.9] |
| Gynaecology Service | 0.0 | [0.0,10.6] |
| Neurology Service | 0.0 | [0.0,12.4] |
| Neurosurgical Service | 2.0 | [0.0,12.6] |
| Ophthalmology Service | 0.0 | [-2.4,8.7] |
| Oral Surgery Service | 0.0 | [0.0,5.1] |
| Plastic Surgery Service | 0.0 | [0.0,9.3] |
| Respiratory Medicine Service | 0.0 | [0.0,14.0] |
| Rheumatology Service | 0.0 | [0.0,12.3] |
| Trauma and Orthopaedic Service | 2.0 | [0.0,11.4] |
| Urology Service | 0.0 | [0.0,12.0] |
|  | **Primary Care Prescription** | |
|  | **Median** | **IQR** |
| Cardiology Service | 2.0 | [-4.6,26.5] |
| Cardiothoracic Surgery Service | 17.3 | [-1.1,34.1] |
| Dermatology Service | 0.0 | [-2.4,15.2] |
| Ear Nose and Throat Service | 2.0 | [-3.5,15.6] |
| Elderly Medicine Service | 3.3 | [-9.8,34.5] |
| Gastroenterology Service | 5.7 | [-0.7,28.9] |
| General Internal Medicine Service | 6.9 | [-1.2,30.5] |
| General Surgery Service | 2.0 | [-3.0,22.4] |
| Gynaecology Service | 0.0 | [-2.8,13.8] |
| Neurology Service | 2.7 | [0.0,27.2] |
| Neurosurgical Service | 8.0 | [-0.2,39.1] |
| Ophthalmology Service | 0.0 | [-8.8,23.8] |
| Oral Surgery Service | 0.0 | [-1.2,5.7] |
| Plastic Surgery Service | 0.0 | [-3.1,15.8] |
| Respiratory Medicine Service | 4.7 | [-2.4,33.0] |
| Rheumatology Service | 4.3 | [0.0,27.1] |
| Trauma and Orthopaedic Service | 4.0 | [-2.7,26.7] |
| Urology Service | 4.0 | [-2.3,28.1] |
|  | **Community** | |
|  | **Median** | **IQR** |
| Cardiology Service | 0.0 | [0.0,0.0] |
| Cardiothoracic Surgery Service | 0.0 | [0.0,0.0] |
| Dermatology Service | 0.0 | [0.0,0.0] |
| Ear Nose and Throat Service | 0.0 | [0.0,0.0] |
| Elderly Medicine Service | 0.0 | [0.0,0.0] |
| Gastroenterology Service | 0.0 | [0.0,0.0] |
| General Internal Medicine Service | 0.0 | [0.0,0.0] |
| General Surgery Service | 0.0 | [0.0,0.0] |
| Gynaecology Service | 0.0 | [0.0,0.0] |
| Neurology Service | 0.0 | [0.0,0.0] |
| Neurosurgical Service | 0.0 | [0.0,0.0] |
| Ophthalmology Service | 0.0 | [0.0,0.0] |
| Oral Surgery Service | 0.0 | [0.0,0.0] |
| Plastic Surgery Service | 0.0 | [0.0,0.0] |
| Respiratory Medicine Service | 0.0 | [0.0,0.0] |
| Rheumatology Service | 0.0 | [0.0,0.0] |
| Trauma and Orthopaedic Service | 0.0 | [0.0,0.0] |
| Urology Service | 0.0 | [0.0,0.0] |
|  | **Emergency Calls** | |
|  | **Median** | **IQR** |
| Cardiology Service | 0.0 | [0.0,0.0] |
| Cardiothoracic Surgery Service | 0.0 | [0.0,0.0] |
| Dermatology Service | 0.0 | [0.0,0.0] |
| Ear Nose and Throat Service | 0.0 | [0.0,0.0] |
| Elderly Medicine Service | 0.0 | [0.0,0.0] |
| Gastroenterology Service | 0.0 | [0.0,0.0] |
| General Internal Medicine Service | 0.0 | [0.0,0.0] |
| General Surgery Service | 0.0 | [0.0,0.0] |
| Gynaecology Service | 0.0 | [0.0,0.0] |
| Neurology Service | 0.0 | [0.0,0.0] |
| Neurosurgical Service | 0.0 | [0.0,0.0] |
| Ophthalmology Service | 0.0 | [0.0,0.0] |
| Oral Surgery Service | 0.0 | [0.0,0.0] |
| Plastic Surgery Service | 0.0 | [0.0,0.0] |
| Respiratory Medicine Service | 0.0 | [0.0,0.0] |
| Rheumatology Service | 0.0 | [0.0,0.0] |
| Trauma and Orthopaedic Service | 0.0 | [0.0,0.0] |
| Urology Service | 0.0 | [0.0,0.0] |
|  | **Secondary** | |
|  | **Median** | **IQR** |
| Cardiology Service | 0.0 | [0.0,7.404] |
| Cardiothoracic Surgery Service | 17.9 | [4.3,33.8] |
| Dermatology Service | 0.0 | [0.0,5.9] |
| Ear Nose and Throat Service | 0.0 | [0.0,6.4] |
| Elderly Medicine Service | 0.0 | [0.0,6.6] |
| Gastroenterology Service | 2.0 | [0.0,11.4] |
| General Internal Medicine Service | 4.0 | [0.0,12.8] |
| General Surgery Service | 2.0 | [0.0,11.1] |
| Gynaecology Service | 0.0 | [0.0,8.7] |
| Neurology Service | 0.0 | [0.0,8.0] |
| Neurosurgical Service | 4.0 | [0.0,12.1] |
| Ophthalmology Service | 0.0 | [0.0,8.0] |
| Oral Surgery Service | 0.0 | [0.0,0.0] |
| Plastic Surgery Service | 2.3 | [0.0,11.9] |
| Respiratory Medicine Service | 5.9 | [0.0,16.0] |
| Rheumatology Service | 0.0 | [0.0,8.4] |
| Trauma and Orthopaedic Service | 4.0 | [0.0,11.8] |
| Urology Service | 2.9 | [0.0,11.9] |
|  | **Mental Health** | |
|  | **Median** | **IQR** |
| Cardiology Service | 0.0 | [0.0,0.0] |
| Cardiothoracic Surgery Service | 0.0 | [0.0,0.0] |
| Dermatology Service | 0.0 | [0.0,0.0] |
| Ear Nose and Throat Service | 0.0 | [0.0,0.0] |
| Elderly Medicine Service | 0.0 | [0.0,0.0] |
| Gastroenterology Service | 0.0 | [0.0,0.0] |
| General Internal Medicine Service | 0.0 | [0.0,0.0] |
| General Surgery Service | 0.0 | [0.0,0.0] |
| Gynaecology Service | 0.0 | [0.0,0.0] |
| Neurology Service | 0.0 | [0.0,0.0] |
| Neurosurgical Service | 0.0 | [0.0,0.0] |
| Ophthalmology Service | 0.0 | [0.0,0.0] |
| Oral Surgery Service | 0.0 | [0.0,0.0] |
| Plastic Surgery Service | 0.0 | [0.0,0.0] |
| Respiratory Medicine Service | 0.0 | [0.0,0.0] |
| Rheumatology Service | 0.0 | [0.0,0.0] |
| Trauma and Orthopaedic Service | 0.0 | [0.0,0.0] |
| Urology Service | 0.0 | [0.0,0.0] |

**Table S2** The amount of additional health service utilisation of patients waiting for treatment. Values represent the number of additional contacts per year.
